# Outcome of preoperative cefazolin use for infection prophylaxis in patients with self-reported penicillin allergy

**DOI:** 10.1186/s12893-023-01931-w

**Published:** 2023-02-08

**Authors:** Laura Michaud, Hope H. Yen, Dale A. Engen, David Yen

**Affiliations:** 1 Department of Surgery, Queen’s University, Kingston, ON Canada; 2 Department of Biostatistics, Queen’s University, Kingston, ON Canada; 3 Department of Anaesthesia and Perioperative Medicine, Queen’s University, Kingston, ON Canada

**Keywords:** Cefazolin, Penicillin allergy, IgE reactions, Non-IgE reactions, Cross reactions

## Abstract

**Background:**

Cephalosporins are the preferred antibiotics for prophylaxis against surgical site infections. Most studies give a rate of combined IgE and non-IgE penicillin allergy yet it is recommended that cephalosporins be avoided in patients having the former but can be used in those with the latter. Some studies use penicillin allergy while others penicillin family allergy rates. The primary goal of this study was to determine the rates of IgE and non-IgE allergy as well as cross reactions to both penicillin and the penicillin family. Secondary goals were to determine the surgical services giving preoperative cefazolin and the types of self reported reactions that patients’ had to penicillin prompting their allergy status.

**Methods:**

All patients undergoing elective and emergency surgery at a University Health Sciences Centre were retrospectively studied. The hospital electronic medical record was used for data collection.

**Results:**

8.9% of our patients reported non-IgE reactions to penicillin with a cross reactivity rate of 0.9% with cefazolin. 4.0% of our patients reported IgE reactions to penicillin with a cross reactivity rate of 4.0% with cefazolin. 10.5% of our patients reported non-IgE reactions to the penicillin family with a cross reactivity rate of 0.8% with cefazolin. 4.3% of our patients reported IgE reactions to the penicillin family with a cross reactivity rate of 4.0% with cefazolin.

**Conclusions:**

Our rate of combined IgE and non-IgE reactions for both penicillin and penicillin family allergy was within the range reported in the literature. Our rate of cross reactivity between cefazolin and combined IgE and non-IgE allergy both to penicillin and the penicillin family were lower than reported in the old literature but within the range of the newer literature. We found a lower rate of allergic reaction to a cephalosporin than reported in the literature. We documented a wide range of IgE and non-IgE reactions. We also demonstrated that cefazolin is frequently the preferred antibiotics for prophylaxis against surgical site infections by many surgical services and that de-labelling patients with penicillin allergy is unnecessary.

## Background

Cephalosporins are the preferred antibiotics for prophylaxis against surgical site infections in many procedures [1, 2]. However, cross-reaction to cephalosporins in patients with a reported penicillin allergy was stated in early studies to be 4 to 18% [3–5]. In addition, the reported allergy to penicillin was in 5–16% of the population [6–11]. This resulted in avoidance of cephalosporins in patients with reported penicillin allergy [12] and prompted use of alternative antibiotics such as Vancomycin and clindamycin [1, 11, 13, 14]. A problem with this approach are the known disadvantages of using Vancomycin including “Vancomycin infusion reaction”, nephrotoxicity and hematologic effects, and decreased effectiveness [15–17]. In addition, clindamycin is less effective at prophylaxis against infection than the use of beta-lactam antibiotics [18–20]. Finally, there is an increased risk of c. difficile, Vancomycin Resistant Enterococcus (VRE), and Methacillin Resistant Staphlococcus Aureus (MRSA) with the use of clindamycin and Vancomycin [9, 21, 22]. Therefore, in an effort to improve antibiotic use, special clinics to de-label penicillin allergies have been started but may not be practical in all settings [7, 23–25].

More recently, the cross reactions to cephalosporins in patients with a penicillin allergy has been reported to be lower than originally thought because of overestimation due to flawed methodologies and since levels of penicillin-specific IgE wane over time [26–30]. Consistent with this, recent studies have reported a lower cross reactivity for cephalosporins in patients with a reported penicillin allergy of 0.2 to 2.4% [26, 28, 31–33]. In addition, the cross reactivity depends on the generation of the cephalosporin [12]. This is because the breakdown of cephalosporin beta-lactam rings do not predictably produce haptens capable of allergenicity and therefore, cross reactivity with penicillins focuses on it’s side chains designated R1 and R2. Although cross reactivity may still exist, it is to a lesser degree with cefazolin because this particular cephalosporin does not share a side chain with any other beta-lactams [28, 33–35].

Most studies give a rate of combined IgE and non-IgE penicillin allergy. Some studies use penicillin allergy while others penicillin family (penicillin, ampicillin, amoxacillin, cloxacillin, nafcillin, piperacillin) allergy rates. In addition, in studies to determine the cross reactivity cefazolin in only patients with self reported non-IgE penicillin allergy, we have previously found no patients to have a reaction when it is given for prophylaxis against surgical site infections in hip and knee replacement surgery, and spine surgery [36, 37]. As such, no rate could be given.

The primary goal of this study was to determine the rates of IgE and non-IgE allergy as well as cross reactions to both penicillin and the penicillin family and to increase the power of our previous studies. Secondary goals were to determine the surgical services giving preoperative cefazolin and the types of self reported reactions that patients’ had to penicillin prompting their allergy status.

## Materials and methods

All patients undergoing elective and emergency surgery at the inpatient and ambulatory care hospitals at a University Health Sciences Centre from July 2015 to December 2015 were retrospectively studied The electronic medical record was used to review the presurgical screening assessment, preoperative history and physical, surgery booking form, O.R. report, intraoperative Anaesthesia record, and recovery room record. From these documents, data was collected concerning preoperative patient reported penicillin or penicillin family allergic reactions, surgical service involved, preoperative antibiotics, and intraoperative or immediate postoperative allergic reactions. IgE and IgE allergy was assumed based on the symptoms reported by the patients. Ethics and consent clearance was obtained from the University Health Sciences & Affiliated Teaching hospitals Research Ethics Board (TRAQ#:6016119).

The data was entered onto a Microsoft Excel version12.3.3 (Redmond, WA) spreadsheet and tabulated using SAS version 9.4 (SAS Institute, Cary, NC). Our rates were compared to the published high and low values using an on-line calculator (https://www.graphpad.com/quickcalcs/contingency1.cfm) to assess statistical significance. When any value in the 2 × 2 table had a frequency of 5 or less, the p-value was generated by the Fisher’s Exact test, otherwise the p-value from the uncorrected Chi-square was utilized. P < 0.05 was considered to be significant.

## Results

**Fig. 1:**
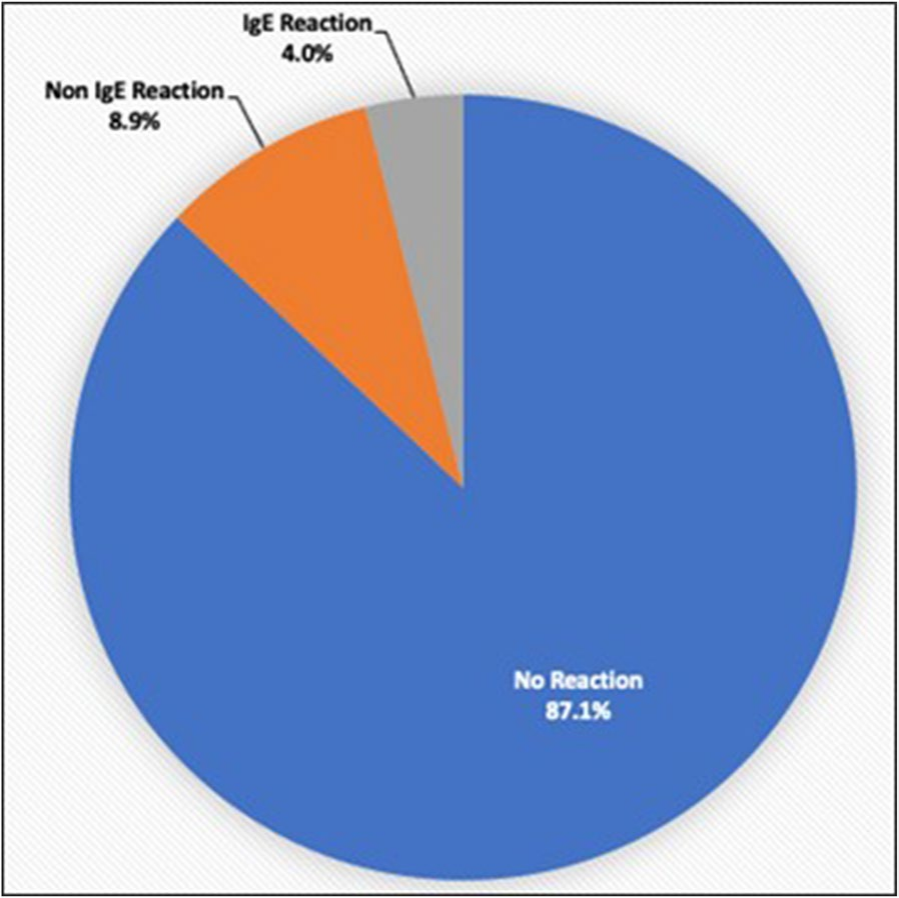
Patients reporting IgE, non-IgE, and no reaction to penicillin

486 of 5433 patients (8.9%) reported non-IgE reactions to penicillin (Fig. 1). 215 of these patients with reported non-IgE reactions got cefazolin and 2 (0.9%) had an adverse reaction. One of these was a patient that reported a rash to penicillin and developed a flat nonpruritis red rash on face and left arm 6.5 h after receiving cefazolin in the recovery room. No treatment and no further mention was made in inpatient notes. The second was an adult patient that reported a childhood allergy to penicillin and became transiently hypotensive with an intraoperative test dose of cefazolin so no further antibiotics were given.

217 of 5433 patients (4.0%) reported IgE reactions to penicillin (Fig. 1). 50 of these patients with reported IgE allergy got cefazolin and 2 (4.0%) had an adverse reaction. One of these was a patient that reported facial swelling to penicillin and needed Benadryl® (Johnson & Johnson Inc.) X 1 for itchiness 4.5 h after getting cefazolin. The second was a patient that reported hives to penicillin and got a chest rash immediately after induction of general anaesthesia that resolved without treatment.

There was a 2/50 (4.0%) cross reactivity between reported IgE allergy to penicillin and cefazolin and a 2/215 (0.9%) cross reactivity between reported non-IgE allergy to penicillin and cefazolin. Therefore, there was a 4/265 (1.5%) cross reactivity between reported allergy to penicillin and cefazolin in this study.

**Fig. 2:**
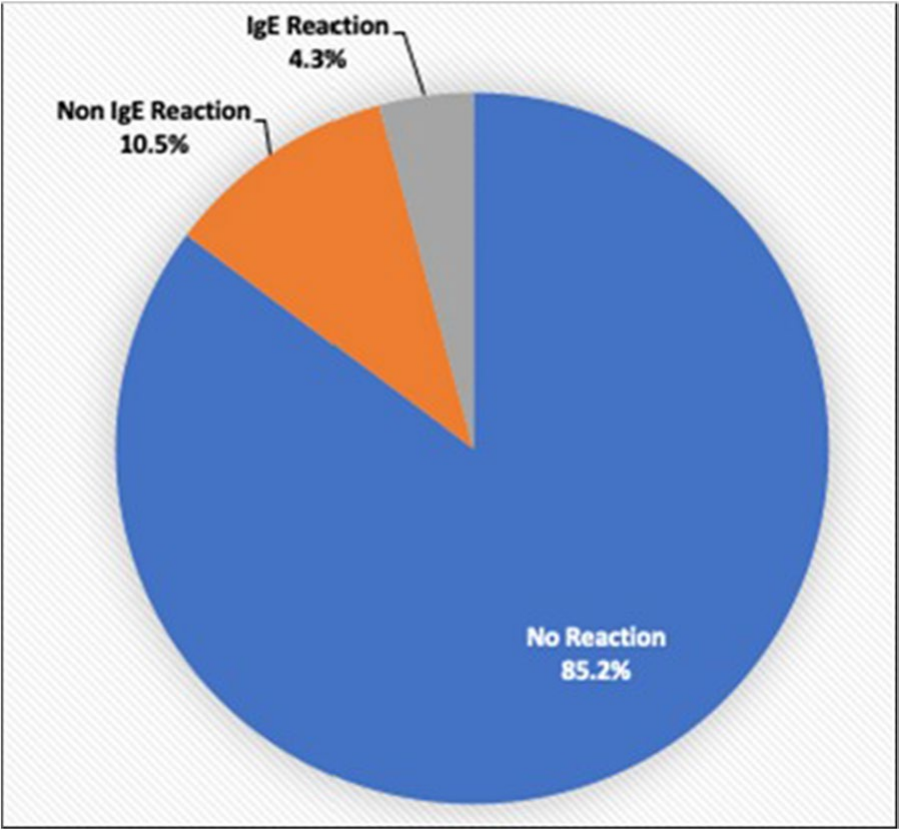
Patients reporting IgE, non-IgE, and no reaction to penicillin family of antibiotics

570 of 5433 patients (10.5%) reported non-IgE reactions to the penicillin family (Fig. 2). 255 of these patients with reported non-IgE reactions got cefazolin and 2 (0.8%) had an adverse reaction (same 2 patients as in the reactions penicillin only).

232 of 5433 patients (4.3%) reported IgE reactions to the penicillin family (Fig. 2). 50 of these patients with reported IgE reactions got cefazolin and 2 (4.0%) had an adverse reaction (same 2 patients as in the reactions penicillin only).

There was a 2/50 (4.0%) cross reactivity between reported IgE reaction to the penicillin family and cefazolin and a 2/255 (0.8%) cross reactivity between reported non-IgE allergy to the penicillin family and cefazolin. Therefore, there was a 4/305 (1.3%) cross reactivity between reported allergy to the penicillin family and cefazolin in this study.

There were a total of 17 out of 5433 patients that had reactions in this study. 12 of these were patients that had an allergic reaction to a cephalosporin (2 with non-IgE reported penicillin allergy-previously described reaction; 2 with IgE reported penicillin allergy-previously described reaction; 7 not reporting an allergy to penicillin [2 itchiness in recovery room, 1 rash intraoperatively, 1 rash in recovery room, 2 hives intraoperatively, 1 hypotension following cefazolin]; 1 reported rash to penicillin given ceftriaxone and Flagyl®(Pfizer) and needed Benedryl® 9.5 h later with no reason given). Four of these were in patients not reporting allergic to penicillin and that did not receive cefazolin (1 transient rash after propofol and before rocuronium; one 3 + puritus despite Benadryl® noted 5.5 h after the general anaesthetic; 1 given Benadryl® X1 5 h postoperatively no reason given; 1 given Decadron®(Merck) X1 3.5 h postoperatively no reason given). One of these was in a patient reporting a rash to ceftazidime and given Vancomycin but stopped because of hypotension and required phenyepherine.

**Fig. 3:**
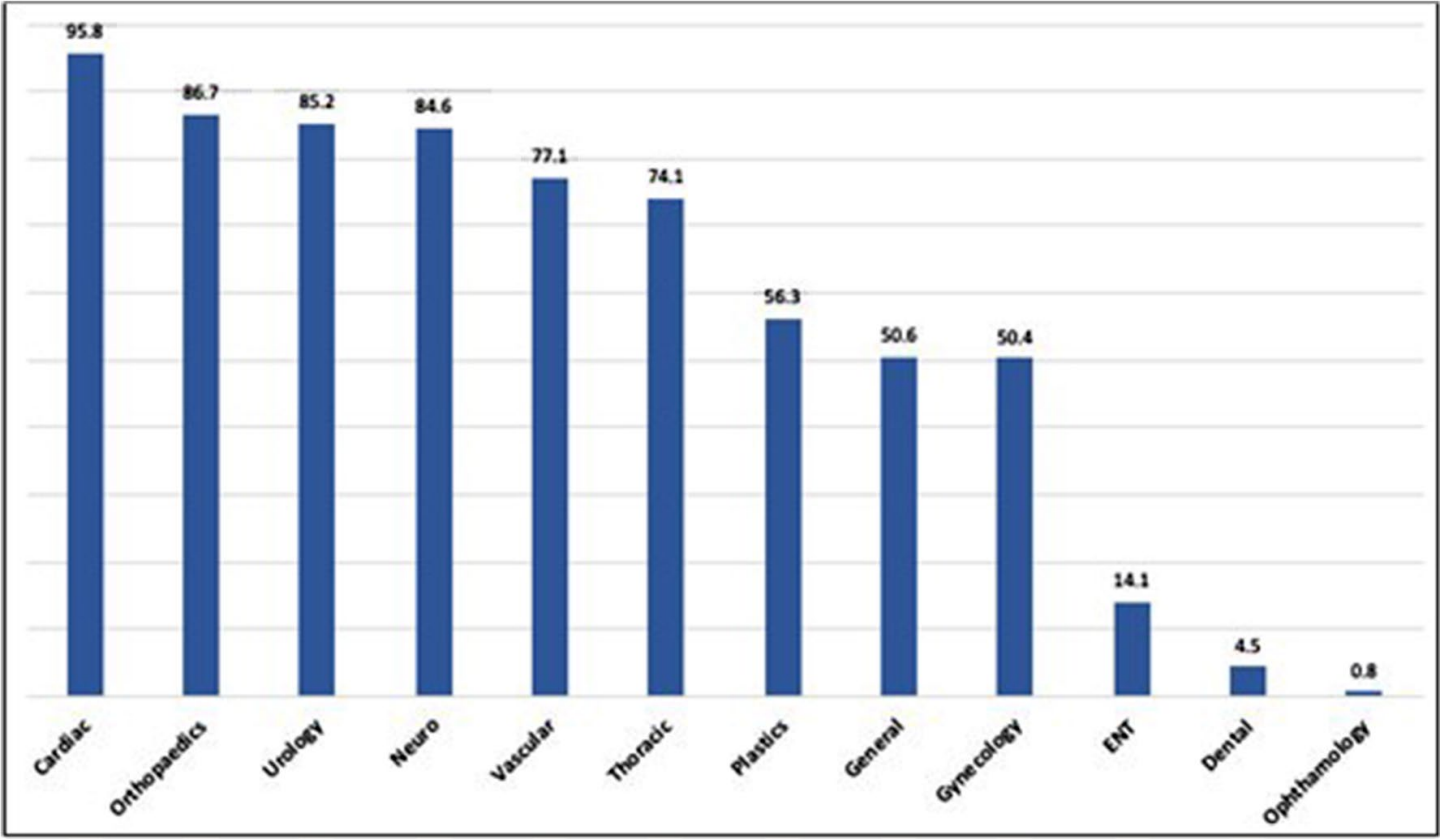
Percentage of their cases given cefazolin by each surgical specialty

203 of 212 Cardiac Surgery cases (95.8%), 1156 of 1334 Orthopaedic cases (86.7%), 317 of 372 Urology cases (85.2%), 181 of 214 Neurosurgery cases (84.6%), 101 of 131 Vascular Surgery cases (77.1%), and 40 of 54 Thoracic Surgery cases (74.1%) were given cefazolin (Fig. 3).

There were no patients that had preoperative penicillin allergy testing. All reported allergies identified as urticaria, immediate airway compromise, hypotension, anaphylaxis, and angioedema were considered IgE-mediated reactions. Rash and unknown were the most commonly reported non-IgE reactions in both penicillin and penicillin family allergy patients (Tables 1, 2).

**Table 1:** Non-IgE patient reported reactions to penicillin

Reported reaction	No. patients	% of Total
Rash	297	61.1
Unknown	73	15.0
GI Upset	45	9.3
Childhood	14	2.9
Other	9	1.9
Family	8	1.6
Ineffective	7	1.4
Empty	7	1.4
Loss of consciousness	3	0.6
Fever	3	0.6
Yeast infection	3	0.6
Skin peels	2	0.4
Elevated heart rate	2	0.4
Dizzy	2	0.4
Dopey	2	0.4
Finger blisters	1	0.2
Can't take, occurred years ago	1	0.2
Built up resistance	1	0.2
Decreased WBC	1	0.2
Jaundice	1	0.2
Sore throat and bloating	1	0.2
Alopecia	1	0.2
GI bleeding	1	0.2
Shakes	1	0.2
Total	486	100.00

**Table 2:** Non-IgE patient reported reactions to penicillin family of antibiotics

Reported reaction	No. patients	% of Total
Rash	342	60.0
Unknown	80	14.0
GI Upset	62	10.9
Other	21	3.7
Childhood	14	2.5
Empty	9	1.6
Family	8	1.4
Ineffective	7	1.2
Loss of consciousness	3	0.5
Fever	3	0.5
Yeast infection	3	0.5
Skin peels	2	0.4
Elevated heart rate	2	0.4
Dizzy	2	0.4
Dopey	2	0.4
Sore throat and bloating	2	0.4
Finger blisters	1	0.2
Can't take, occurred years ago	1	0.2
Built up resistance	1	0.2
Decreased WBC	1	0.2
Jaundice	1	0.2
Alopecia	1	0.2
GI bleeding	1	0.2
Shakes	1	0.2
Total	570	100.00

## Discussion

As recommended in the literature, we found cefazolin frequently the preferred antibiotics for prophylaxis against surgical site infections by many surgical services. Cardiac surgery (95.8%), Orthopaedics (86.7%), Urology (85.2%), Neurosurgery (84.6%), Vascular Surgery (77.1%), and Thoracic Surgery (74.1%) had the highest percentage per case use of cefazolin (Fig. 3).

Most studies give a rate of combined IgE and non-IgE penicillin allergy. However, recommendations for use of cephalosporins depends on the type of allergy to penicillin. Specifically, Pichichero recommends that if a patient experienced a reaction to a penicillin or cephalosporin that was not IgE mediated and was not serious, it is safe to administer repeated courses of that antibiotic and related antibiotics. Only IgE mediated reactions are likely to become more severe with time and to result in anaphylaxis [26]. This recommendation and because our previous work demonstrated the safety of cefazolin use only in non-IgE penicillin allergy, made us unsure whether the same rates of reactions would apply to IgE penicillin allergy patients. Therefore, we separated both the rates of penicillin allergy and the cross reactions into patients reporting IgE and non-IgE reactions. With this distinction, we found that 50 patients with reported IgE allergy got cefazolin and 2 (4.0%) had an adverse reaction. Both adverse reactions were non-IgE (1 itchiness and 1 chest rash) but our numbers are so small that no conclusions can be made about the safety of cefazolin use in patients reporting an IgE allergy to penicillin.

Some studies use penicillin allergy while others penicillin family allergy rates. We found a 12.9% (8.9% IgE and 4.0% non-IgE) reported rate of penicillin allergy versus 14.8% (10.5% IgE and 4.3% non-IgE) for the penicillin family. Both are within the range reported in the literature where there is a high of 15.6% and a low of 5.0% (Table 3). However, we prefer the use of the penicillin family for inclusion as a penicillin allergy to be consistent with penicillin allergy testing which uses an oral challenge with amoxacillin.

**Table 3:** Study vs. literature rates for penicillin allergy, cross reactivity, and cephalosporin allergy

Group	Our rate	Literature	P-value	Reference source
Penicillin allergy	703/5433 = 12.9%	High 295/1893 = 15.6%	P = 0.07	20. Lee CE, Arch Intern Med 2000;160:2819–22
		Low 1821/3643 = 5.0%	P < 0.0001	8. Borch JE., Basic & Clinical pharmacology & Toxicology 2006; 98:357–362
Cross reaction of penicillin and cephalosporin	4/265 = 1.5% with cefazolin	Old high 2/11 = 18.2% with cephalothin	P = 0.02	7. Thoburn R, JAMA 1966;198:345
		Old low 3/74 = 4.1% with cefazolin	P = 0.18	4. Petz LD. J Infect Dis 1978;137(Suppl):S74-S9
		New high 1/41 = 2.4% with cephalosporin	P = 0.52	55. Novalbos A, Clin Exp Allergy 2001;31: 438–43
		New low 1/606 = 0.2% with cephalosporin	P = 0.03	49. Daulat S, J Allergy Clin Immunol 2004;113: 1220–2
Cephalosporin allergy	12/2873 = 0.4%	High 10/473 = 2.1%	P < 0.0001	Bigby M, JAMA. 1986; 256(24):3358–3363
		Low 7035/949323 = 0.7%	P < 0.05	Macy E, J Allergy Clin Immunol 2015;135: 745–752.e5

We found a 1.5% (4.0% IgE and 0.9% non-IgE) cross reactivity between reported allergy to penicillin and cefazolin and 1.3% (4.0% and 0.8%) cross reactivity between penicillin family and cefazolin. Both are lower than the old literature high of 18.2% and low of 4.1%. Both are within the newer literature high of 2.4% and low of 0.2% (Table 3).

The frequency of allergic reactions to cephalosporins is 0.7–2.1% [12, 26, 38, 39]. We found 12/2873 (0.4%) of patients had an allergic reaction to a cephalosporin. This is lower than reported in the literature where there is a high of 2.1% and low of 0.7% (Table 3). This may be due to our small numbers but is agreement with the literature that in addition to cross reactivity to penicillin, an allergy to cefazolin itself is an important risk factor for a reaction when it is given as prophylaxis against surgical site infection [26]. This is logical when considering the role of chemical structure in determining the risk of cross-reactivity and specifically, that the incidence of cross reactivity with cephalosporins in penicillin-allergic patients varies with the chemical side chain similarity of the cephalosporin to penicillin or amoxicillin [28, 33, 34] with cefazolin not sharing a side chain with any other beta-lactams [35].

We acknowledge that penicillin testing is necessary to truly distinguish between IgE and non IgE allergy but have found practical difficulties accessing this as a routine preoperative service. Therefore, our goal was to provide clinical guidance based on patient reported allergy.

While de-labelling may be useful in the infection treatment setting, we believe that it is not necessary in patients requiring preoperative antibiotic prophylaxis because of our 305 patients that reported a penicillin family allergy and got cefazolin, 4 had a reaction. Three of these were non-IgE (2 rashes requiring no treatment and 1 itchiness treated with Benedryl®) which could have safely been given cefazolin [12, 26]. The fourth of these was IgE (hypotension with a test dose) which would not have been predicted because the allergy was a reported as a childhood allergy that should have diminished by the time of surgery [29, 30].

This study was limited by its retrospective nature, which may have affected the accuracy of data recorded. Reactions could have been missed or alternatively, clinical signs and symptoms of an allergic reaction could have been inappropriately attributed to the administration of antibiotics when in fact they were due to some other medication or condition. Examples of this latter possibility is that of our 17 patients with reactions, 4 got no antibiotics yet 1 had a transient rash after propofol and before rocuronium; 1was given Benadryl® X1, 5 h postoperatively with no reason given; 1 was given Decadron® × 1, 3.5 h postop with no reason given; 1 had 3 + pruritis despite Benadryl® noted 5.5 h after general anaesthesia.

A further limitation is that we looked for reactions only up to the time that the patients were recovered from surgery, typically < 24 h and did not address delayed reactions. Although we recorded non-IgE reactions (Tables 1, 2), IgE mediated reactions are what we were focused on because of their clinical importance and would be expected to have manifested during this time period [12, 40].

## Conclusions

We met our primary goals of separating the rates of IgE and non-IgE allergy and cross reactions to penicillin and the penicillin family. We found that the combined rates of combined IgE and non-IgE reactions for both penicillin and penicillin family allergy was within the range reported in the literature. Our rate of cross reactivity between cefazolin and combined IgE and non-IgE allergy both to penicillin and the penicillin family were lower than reported in the old literature but within the range of the newer literature. We demonstrated a lower rate of allergic reaction to a cephalosporin than reported in the literature. We also determine that cefazolin is frequently the preferred antibiotics for prophylaxis against surgical site infections by many surgical services and that de-labelling is not necessary.

## Data Availability

The datasets used and/or analysed during the current study are available from the corresponding author on reasonable request.

## Abbreviations

VRE: Vancomycin Resistant Enterococcus
MRSA: Methacillin Resistant Staphlococcus Aureus
